# Complexing Methylene Blue with Phosphorus Dendrimers to Increase Photodynamic Activity

**DOI:** 10.3390/molecules22030345

**Published:** 2017-02-23

**Authors:** Monika Dabrzalska, Anna Janaszewska, Maria Zablocka, Serge Mignani, Jean Pierre Majoral, Barbara Klajnert-Maculewicz

**Affiliations:** 1 Department of General Biophysics, Faculty of Biology and Environmental Protection, University of Lodz, Pomorska 141/143, 90-236 Lodz, Poland; rozanek.monika@gmail.com (M.D.); ankuj@biol.uni.lodz.pl (A.J.); 2 Centre of Molecular and Macromolecular Studies, Polish Academy of Sciences, Sienkiewicza 112, 90-363 Lodz, Poland; zabloc@cbmm.lodz.pl; 3 Laboratoire de Chimie et de Biochimie Pharmacologiques et Toxicologique, Université Paris Descartes, PRES Sorbonne Paris Cité, CNRS UMR 860, 45 Rue Des Saints Pères, 75006 Paris, France; serge.mignani@parisdescartes.fr; 4 Laboratoire de Chimie de Coordination CNRS, 205 Route de Narbonne, 31077 Toulouse CEDEX 4, France; jean-pierre.majoral@lcc-toulouse.fr; 5 Institut National Polytechnique de Toulouse, Université de Toulouse, UPS, 31077 Toulouse CEDEX 4, France; 6 Institut für Polymerforschung Dresden e.V., Hohe Strasse 6, 01069 Dresden, Germany

**Keywords:** methylene blue, phosphorus dendrimer, nanocarrier, photosensitizer, photodynamic therapy, phototoxicity, basal cell carcinoma

## Abstract

The efficiency of photodynamic therapy is limited mainly due to low selectivity, unfavorable biodistribution of photosensitizers, and long-lasting skin sensitivity to light. However, drug delivery systems based on nanoparticles may overcome the limitations mentioned above. Among others, dendrimers are particularly attractive as carriers, because of their globular architecture and high loading capacity. The goal of the study was to check whether an anionic phosphorus dendrimer is suitable as a carrier of a photosensitizer—methylene blue (MB). As a biological model, basal cell carcinoma cell lines were used. We checked the influence of the MB complexation on its singlet oxygen production ability using a commercial fluorescence probe. Next, cellular uptake, phototoxicity, reactive oxygen species (ROS) generation, and cell death were investigated. The MB-anionic dendrimer complex (MB-1an) was found to generate less singlet oxygen; however, the complex showed higher cellular uptake and phototoxicity against basal cell carcinoma cell lines, which was accompanied with enhanced ROS production. Owing to the obtained results, we conclude that the photodynamic activity of MB complexed with an anionic dendrimer is higher than free MB against basal cell carcinoma cell lines.

## 1. Introduction

Photodynamic therapy (PDT) relies on topical or systemic administration of a photosensitizer (PS), which should be toxic only after irradiation with a certain wavelength of visible light. Hence, the PS, even when accumulated in normal cells, should not exhibit a toxic effect; so-called ‘dark toxicity’. The mechanism of a photodynamic action is based on energy transfer between an excited triplet state photosensitizer and a cell environment in the presence of oxygen. The photosensitizer can generate reactive oxygen species, such as a hydroxyl radical or a superoxide anion, which is the Type I mechanism, or generate singlet oxygen molecules, which is the Type II mechanism of PDT [1].

Although the photodynamic therapy is an attractive way to treat cancer, it has some drawbacks that limit its usefulness. Mainly, it is the unwanted dark toxicity of photosensitizers, inefficient cellular uptake of PS in cancer cells or its hydrophobicity. In addition, PDT is generally limited to local treatment because of the need to activate the photosensitizer with light accessing a tumor. However, the topical character is also an advantage of PDT over conventional chemotherapy because of the desired site-specific toxicity of the photosensitizer. Unfortunately, the selectivity of photosensitizers is still a challenge in clinical PDT [2,3]. The problems associated with photosensitizers may be resolved by designing drug delivery systems for photosensitizers, similarly as in the case of chemotherapeutics. Nanoparticles may enhance the solubility of photosensitizers, and improve the cellular uptake and specificity due to a nanometric size and controllable properties, such as charge or hydrophilicity [4].

Among the variety of known nanoparticles, dendrimers possess remarkable potential as carriers of photosensitizers. Dendrimers are globular, hyperbranched polymers that consist of a central core, repeated branches (called ‘dendrons’) and terminal groups. The more layers of monomers that are attached, the higher the generation of the dendrimer. A photosensitizer molecule may be centered at the core of the dendrimer, attached to the internal groups, encapsulated in the inner cavities, or attached to the terminal groups on the surface. Complexation of a drug and a dendrimer may be driven by covalent or non-covalent interactions. The unique dendritic architecture makes dendrimers ideal candidates to transport active molecules. Dendrimers have been reported to improve the photodynamic efficacy of several photosensitizers [5,6]. Polyamidoamine (PAMAM) dendrimer conjugated with a near-infrared sensitive photosensitizer and a targeting peptide showed high phototoxicity against a HER-2 positive cell line and in a xenograft animal tumor model [7]. Dendrimers are also being investigated in more complex drug delivery systems. For example, loading an anionic dendrimer possessing a phthalocyanine molecule in the core into polymeric micelles enhanced a photochemical effect and phototoxicity in vitro [8]. The positive effect of dendrimers as photosensitizer carriers has been also reported in case of protoporphyrin [9] and aminolaevulinic acid [10].

Methylene blue (MB) belongs to the phenothiazinum family of compounds and it has been widely used as a histological dye. The methylene blue absorption spectrum consists of two peaks, 664 nm and 590 nm, representing MB monomers and dimers, respectively. The shape of the MB absorption spectrum depends on the concentration and the ratio of monomers/dimers of MB. Methylene blue has been also approved to treat methemoglobinemia, which is a condition characterized by the increase of an oxidized form of hemoglobin (methemoglobin) that does not have the ability to bind oxygen molecules. MB is an attractive photosensitizer because of a number of reasons, i.e., it is a hydrophilic, low-cost compound, it has strong absorption band in the red spectral region, and a high quantum yield of singlet oxygen generation [11]. MB was reported as an effective photosensitizer in PDT against melanoma [12]. Recently, Samy and colleagues conducted a clinical trial using free MB and liposomal MB in patients with basal cell carcinoma (BCC) lesions. Results of this study indicated that photodynamic therapy using MB was an effective method for treatment of BCC tumors with very good cosmetic outcomes [13].

However, the clinical use of MB can be hampered because MB is prone to aggregation and to rapid chemical alterations in a biological environment. After systemic administration, MB molecules usually accept electrons from NADH/NADPH, which reduces MB to leukomethylene blue. MB can also be reduced by thiazine dye reductase or NADH/NADPH dehydrogenases. Importantly, the reduced form of MB exhibits negligible photodynamic efficiency [14,15]. It has been earlier demonstrated that the use of carriers for MB molecules, such as silica nanocomposites [16] or calcium phosphate nanoparticles [17], may improve its photodynamic efficacy in vitro and protect the PS from the enzymatic reduction.

In our previous studies, we evaluated whether the anionic dendrimer might be a carrier of MB molecules and, thus, we investigated the interactions between methylene blue and anionic phosphorus dendrimers of a second generation possessing 24 carboxylic terminal groups (1an). We used Fourier transform infrared spectroscopy (FTIR) and spectrophotometry to establish the possible interactions. Obtained results confirmed that the anionic dendrimer formed a complex with methylene blue. Based on the analysis of the changes in the MB absorption spectrum we concluded that the stoichiometry of the MB:dendrimer (MB-1an) complex ranged from 9:1 to 10:1. This study also showed that interactions between the cationic dye and the anionic dendrimer were not only electrostatic, but also via π stacking. The obtained complexes were stable up to 24 h. The formation of a stable complex allowed proposing an anionic phosphorus dendrimer as a potential candidate for a MB carrier in PDT [18,19]. In this paper, we aim to further evaluate this complex in comparison with free MB, considering the singlet oxygen generation and in vitro studies. We chose murine basal cell carcinoma (BCC) cell lines as an in vitro model for this study because BCC is one of the most common cancers in the human population. BCC tumors grow slowly and usually do not metastasize, but without a proper treatment, it may cause severe disfigurement [20,21]. BCC lesions are suitable for a topical photodynamic treatment and as it was mentioned above, MB has already been proved as an efficient PS against BCC lesions in a clinical trial [13].

## 2. Results

**Figure 1:**
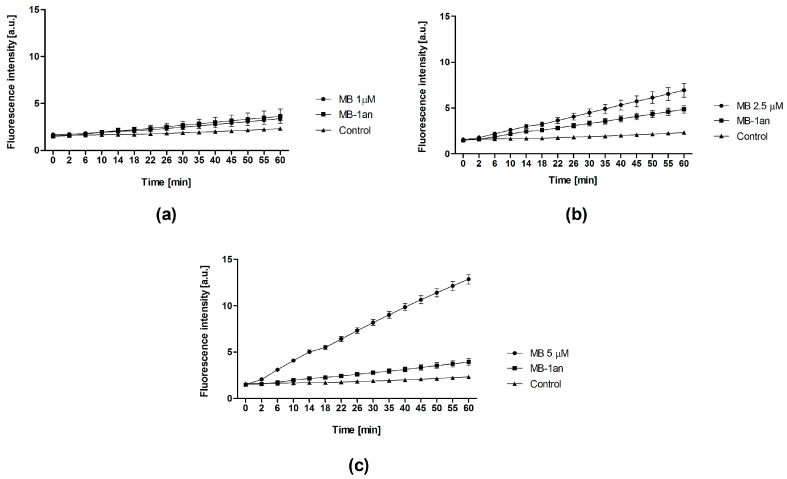
The fluorescence intensity of the singlet oxygen sensor green (SOSG) probe in the presence of different concentrations of methylene blue (MB) and methylene blue-dendrimer complex (MB-1an): 1 µM (**a**); 2.5 µM (**b**); and 5 µM (**c**) versus the time of irradiation.

Firstly, we compared singlet oxygen production of free MB and MB-1an in an aqueous medium. We used a highly selective singlet oxygen sensor green (SOSG) probe, which originally exhibits weak blue fluorescence, but in the presence of singlet oxygen SOSG fluorescence intensity increases and shifts to longer wavelengths. As it can be seen in Figure 1, the fluorescence of SOSG increased over the time of irradiation in the presence of MB-1an and MB. For the lower concentration, no significant differences were observed between MB and MB-1an. However, the values of SOSG fluorescence intensity were lower in the presence of MB-1an than in the presence of free MB at concentrations of 2.5 and 5 µM.

**Figure 2:**
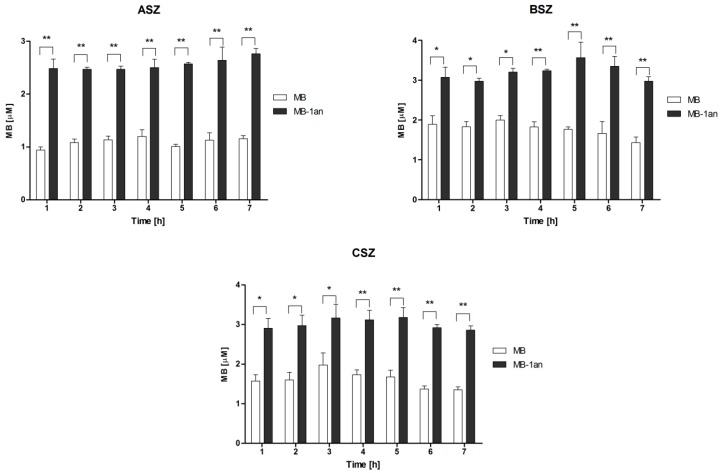
Cellular uptake of MB and MB-1an for ASZ, BSZ, and CSZ cell line, after incubation times from 1 up to 7 h. The concentration of MB was equal to 5 µM and the dendrimer concentration was 1 µM. Molar ratio of the complex (MB:1an = 5:1),. * *p* < 0.05, ** *p* < 0.001.

In order to verify the cellular uptake of free MB and MB-1an, cells were lysed and the absorbance of MB at 664 nm was measured. For this experiment, we chose the concentration of MB (5 µM) at which we observed the highest difference in singlet oxygen production between MB and MB1an in aqueous medium. The cellular uptake was calculated as a concentration of MB based on the standard curve of MB absorbance. Figure 2 depicts the percent of cellular uptake for MB and MB-1cat, where the concentration of MB was 5 µM and the MB:1an ratio was 5:1, for incubation times from 1 to 7 h. The results are presented for three representative BCC cell lines, i.e., ASZ, BSZ, and CSZ.

**Figure 3:**
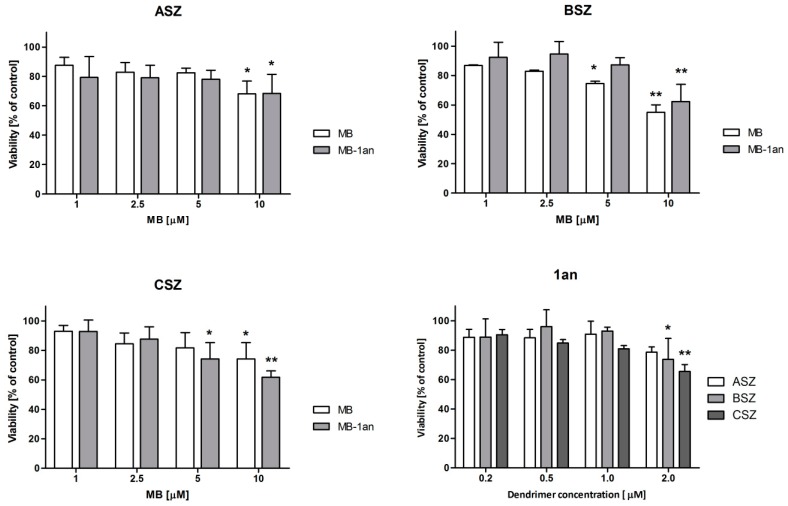
Viability of ASZ, BSZ, and CSZ cell lines after the treatment with MB, MB-1an and 1an without irradiation (dark toxicity). The concentrations of MB varied from 1 to 10 µM, whereas the dendrimer concentration used ranged from 0.2 to 2.0 µM. * *p* < 0.05, ** *p* < 0.01.

In all three cell lines the percentage of MB-1an taken up by the cells was higher than that of a free MB. Interestingly, the cellular uptake of MB and MB-1an did not show strong dependency on the time of incubation. In the case of the ASZ cell line, the uptake of MB and MB-1an by the cells was lower than in BSZ and CSZ cell lines. The uptake in the ASZ cell line was between 0.9 and 1.15 µM in the case of MB, and between 2.4 and 2.76 µM for MB-1an. In both cell lines, the highest uptake of MB (1.9 µM) was after three hours of incubation. After 5 h, MB-1an uptake by BSZ cell line was 1.7 µM and in CSZ cell line it was 1.76 µM. In BSZ cell line, after 5 h, MB-1an was taken up by the cells two-fold higher than free MB. The 5 h incubation time was chosen for further experiments. Before any experiments involving irradiations, it is crucial to check whether the designed delivery system cause any toxic effect in the dark. Also, it is important to evaluate the effect of the nanocarrier (in this study anionic dendrimer 1an), because the carrier itself should not induce any cytotoxicity. Hence, we checked the dark toxicity of free MB, MB-1an and 1an. The effects of tested compounds on three BCC cell lines (ASZ, BSZ, and CSZ) are depicted in Figure 3.

**Figure 4:**
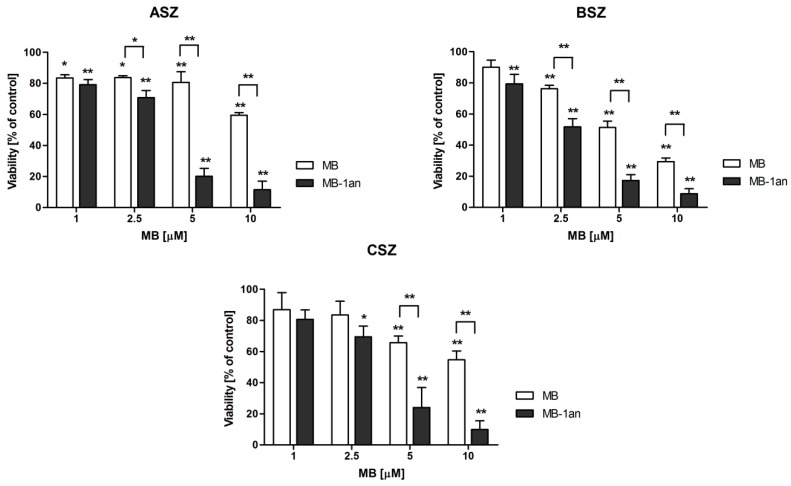
Viability of ASZ, BSZ and CSZ cell lines after the treatment with different concentrations of MB and MB-1an after the 30 min irradiation. The light source was a Q.Light Pro Unit (Q.Products AG, Rorschach, Switzerland) lamp. * *p* < 0.05, ** *p* < 0.001.

Only MB at the concentration of 10 µM was found to be slightly toxic, both in the case of the free MB and the MB-1an complex. The decrease in cell viability was observed in all three cell lines. Moreover, statistically significant viability decrease was confirmed for 5 µM MB in the BSZ cell line and 5 µM MB-1an in the CSZ cell line. In the case of 1an dendrimer, it also caused a slight decrease in viability of BSZ and CSZ cell lines at a concentration of 2 µM. The viability of the cells in these cases was, however, close to 80% and the viability above 80% is considered as non-toxic according to ISO 10993-5:2009 [22]. Taking these results into account, we performed the cytotoxicity tests of MB and MB-1an after irradiation. Figure 4 shows the viability of ASZ, BSZ, and CSZ cell lines after the incubation with MB and MB-1an and 30 min of irradiation with red light.

**Figure 5:**
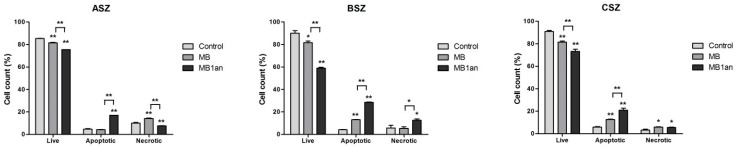
The percentage of live, apoptotic and necrotic cells in ASZ, BSZ, and CSZ cell lines after the irradiation with MB and MB-1an. * *p* < 0.05, ** *p* < 0.001.

The lowest concentration of MB reduced cell viability to approximately 80% and, therefore, we considered this concentration of MB and MB-1an as non-phototoxic. However, a statistically significant difference between the toxicity of MB and MB-1an was found in ASZ and BSZ cell lines. Importantly, the viability of the BSZ cell line decreased below 60% and the MB-1an complex toxicity was significantly higher than that of MB (see Supplementary Materials). The strongest effects were observed in the case of MB and MB-1an at a concentration of 5 µM at which cell viability was significantly decreased, demonstrating the obvious photodynamic activity of MB and MB-1an. In the ASZ cell line MB did not cause significant toxic effects, while MB-1an decreased the cell viability to 20%. In the case of BSZ and CSZ cell lines, MB-1an showed also higher phototoxic effect. The viability of the BSZ cell line after the irradiation with MB-1an was 17% and CSZ cell line showed viability of 24% (see Supplementary Materials). As it is shown in Figure 4, the MB-1an complex caused significantly higher phototoxicity than free MB above a concentration of 1 µM. Based on the observed cytotoxicity, we checked the apoptotic and necrotic cell fractions after the irradiation of BCC cell lines with MB and MB-1an. The concentration of MB used in this experiment was 2.5 µM as at this concentration significant differences in toxicity were observed, but still more than 50% of the cells were alive. Figure 5 shows the percentage of live, apoptotic, and necrotic cells in BCC cell lines.

There was a statistically significant difference between the percent of live BCC cells. More apoptotic and necrotic cells were found after the treatment with MB-1an, although the difference between MB and MB-1an was statistically significant in the case of the percent of apoptotic and necrotic (except for the CSZ cell line) cells. In the case of BSZ cell line, fractions of apoptotic and necrotic cells were the highest and equaled to 28.5% and 12.6%, respectively.

**Figure 6:**
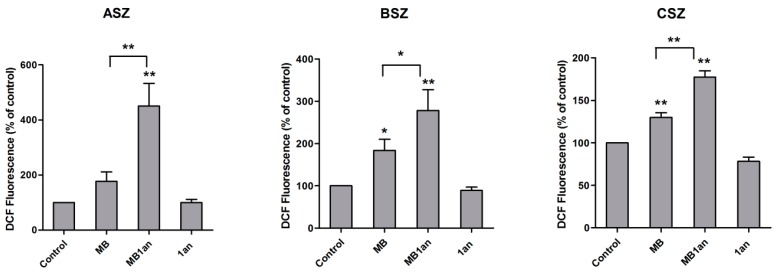
Reactive oxygen species production in ASZ, BSZ, and CSZ cell lines upon irradiation and incubation with MB, MB-1an and 1an presented as DCF fluorescence (% of control). The concentration of MB was 5 µM and the 1an concentration was 1 µM. * *p* < 0.05, ** *p* < 0.001.

As it was shown that MB:1an phosphorus dendrimer complex at 5:1 ratio was transported to BCC cells more effectively and showed higher phototoxicity against BCC cells, it was important to compare the level of reactive oxygen species (ROS) generation after irradiation for free and complexed MB. The production of ROS was monitored by fluorescence intensity of molecular probe H2DCFDA and calculated as a percent of the control which was 100%. Figure 6 depicts the level of ROS after the irradiation of BCC cells after incubation with MB, MB-1an, and 1an.

1an was used in order to check whether the dendrimer generates ROS in cells since it caused the decrease in singlet oxygen production by MB in aqueous environment. In this experiment, MB was also used in a concentration of 5 µM. The highest difference between ROS level generated by MB and MB-1an was in ASZ cell line. MB-1an generated more than twice ROS than free MB. These results are consistent with cytotoxicity results of MB-1an in ASZ cell line where the difference between cell viability of cells irradiated with 5 µM MB and MB-1an was also the most significant. MB-1an generated ROS more efficiently in BSZ and CSZ cell lines. However, the 1an dendrimer did not produce more ROS in comparison with the control.

## 3. Discussion

Current cancer treatments need to meet the challenge of improving its efficiency and limiting side effects. The limitations of currently used drugs also affect photodynamic therapy, which is a good therapeutic tool for local treatment of various diseases [2,23]. Nanoparticles, and particularly dendrimers, are attractive carriers of small molecules, including photosensitizers [5]. In this study, we present the evidence that anionic phosphorus dendrimer is an effective carrier of methylene blue and improves its photodynamic activity in basal cell carcinoma cell lines.

In our previous papers, we showed that two phosphorus dendrimers formed stable complexes with hydrophilic photosensitizers [18,19]. Here, our aim was to evaluate photodynamic activity of methylene blue-anionic dendrimer complex.

There are two known mechanisms of photodynamic action of photosensitizers: Type I, which is a generation of ROS, and Type II—the generation of singlet oxygen. Methylene blue is characterized by high quantum yield of singlet oxygen generation and therefore, we firstly checked the production of singlet oxygen by MB and MB-1an in an aqueous environment. The obtained results showed that both free MB and MB-1an effectively generated singlet oxygen as a function of irradiation time. The MB-1an complex, however, generated significantly less singlet oxygen. Thus, the complexation of MB influenced its efficiency of the singlet oxygen production in the aqueous environment. The main factor diminishing the photodynamic efficiency of photosensitizers is aggregation. Methylene blue tends to aggregate in the aqueous medium; in our previous study [19] we confirmed the interaction between MB and anionic dendrimer, but we did not observe the aggregation of MB. Moreover, we showed that MB might interact not only with the surface of the dendrimer, but also with the dendrimer’ cavities. MB molecules interacting with the interior groups of the anionic dendrimer and buried in a hydrophobic part of dendrimer might be less accessible to oxygen molecules, thus, resulting in a reduced amount of singlet oxygen generated by the MB-1an complex. Similarly to our results, lower singlet oxygen production by MB complexed with nanoparticles was reported in the case of MB-coated silica nanoparticles [16].

Confirming the ability of the complex to generate singlet oxygen in aqueous environment, we next investigated the cellular uptake of free MB and MB-1an in three murine basal cell carcinoma cell lines: ASZ, BSZ, and CSZ. The three cell lines were derived from mice bearing different mutations characteristic to human BCC tumors. The tumors were developed under different conditions and, therefore, there are genetic differences between the cell lines. Thus, these three murine cell lines are good biological models of basal cell carcinoma [24]. Our study showed that MB-1an was taken up by all three cell lines more efficiently than free MB. However, the cellular uptake of both MB and MB-1an did not show dependency with time. This result may be interpreted that both compounds were delivered rapidly to the cells. In the case of methylene blue, it has been proven that MB easily crosses cell membrane due to cationic charge and interactions with the negatively-charged membrane [11,25]. The 1an dendrimer possesses anionic surface groups and it is known that the anionic charge prevent from the interaction with negatively charged membranes making the anionic dendrimers, in most cases, non-toxic in vitro. Nevertheless, the efficient cellular uptake was reported in the case of anionic dendrimers. Studies showed that anionic PAMAM dendrimer possessing carboxyl terminal groups was effectively taken through caveolae-mediated endocytosis [26]. The highest difference between the uptake of MB-1an and MB was in BSZ cell line after 5 h. In ASZ and CSZ cell lines the uptake was also respectively high and, therefore, we chose a five-hour incubation time as optimal for further experiments.

When considering any new system carrying photosensitizers, two elements need to be checked. The first is a cytotoxicity of the carrier itself and the latter is the dark toxicity of the photosensitizer-carrier complex. Thus, we firstly evaluated the cytotoxicity of free MB, MB-1an, and 1an in BCC lines. As it was mentioned above, anionic dendrimer, due to its charge, is not supposed to interact with the negatively-charged cell membrane and cause membrane disruption. Our results confirmed this. The dark toxicity results showed that only the highest used concentration of the compounds, including dendrimer, influenced the viability of the cells, yet the viability was above 70%. Moreover, dark toxicity of the MB-1an complex was not higher than for free MB. These promising results led us to check the key property of MB-1an complex, i.e., its phototoxicity. The experiments showed that MB and MB-1an were able to induce toxic effect in BCC after the irradiation with broad spectrum lamp equipped with a red filter. Importantly, the MB-1an complex showed significantly higher phototoxicity than free MB at 2.5 µM concentration in ASZ and BSZ cell lines. The most pronounced difference in phototoxicity of MB-1an and MB was at the concentration of 5 µM in all three cell lines. These results are in agreement with the cellular uptake studies, suggesting that higher toxicity of the MB-1an complex was due to enhanced uptake by the cells. Similarly, higher photodynamic efficiency was reported for MB encapsulated in anionic microgel [27] and silica nanocomposites [16]. In both cases the higher toxicity was also connected to enhanced uptake. Knowing that complexation did not have a beneficial effect on the generation of singlet oxygen in the aqueous medium, we decided to check its impact on the production of reactive oxygen species in cells. We showed that MB generated ROS in all BCC lines. For all lines MB-1an caused much higher production of ROS. This effect was the most pronounced for ASZ cells. In PDT, both mechanisms (Type I and Type II) may occur simultaneously. Importantly, the ratio between these two mechanisms depends on several factors, such as the type of photosensitizer, the amount of oxygen, and the available substrates [28]. It is interesting that the complex generated less singlet oxygen than free MB in aqueous medium and, contrarily, the complex showed enhanced ROS production in cells. MB has shown the ability to localize in mitochondria, however, some study shows that the MB accumulated in mitochondria with high proton potential may form dimers and, therefore, generate less singlet oxygen [25]. Since the measurements of singlet oxygen was performed in a model aqueous environment, and the ROS assays were done in a cellular system, it is not possible to compare these two experiments directly. The discrepancy between the singlet oxygen production and ROS generation may be caused by the differences in experimental conditions and, therefore, in the amount of available substrates for the photophysical reactions in these two systems.

This finding is consistent with cytotoxicity results, where also for ASZ cells the greatest effect was observed. MB photodynamic activity was reported to induce apoptosis in association with down-regulation of anti-apoptotic proteins, reduction of mitochondrial membrane potential (MMP), increase of phosphorylation of the mitogen-activated protein kinase (MAPK), and generation of reactive oxygen species (ROS) in several cell lines [12,29,30]. Our results indicated a high fraction of apoptotic, but also of necrotic cells, suggesting that MB and MB-1an mediated photodynamic effect induces both apoptosis and necrosis in BCC lines.

To sum up, MB-1an complex is an efficient photosensitizing agent against three basal cell carcinoma cell lines. MB-1an complex showed enhanced cellular uptake by BCC cell lines. What is important, the dendrimer as a carrier and MB-1an complex did not show significant influence on the cell viability in the dark. However, after the irradiation with a red-filtered broad spectrum lamp, the MB-1an complex showed significantly higher phototoxicity, accompanied with a respective increase of ROS generation. Therefore, the MB-1an complex possesses therapeutic potential in photodynamic therapy. Taking into account the obtained results, further studies on the cellular trafficking, photodynamic action, and cell death mechanism are needed.

## 4. Materials and Methods

### 4.1. Materials

**Scheme 1:**
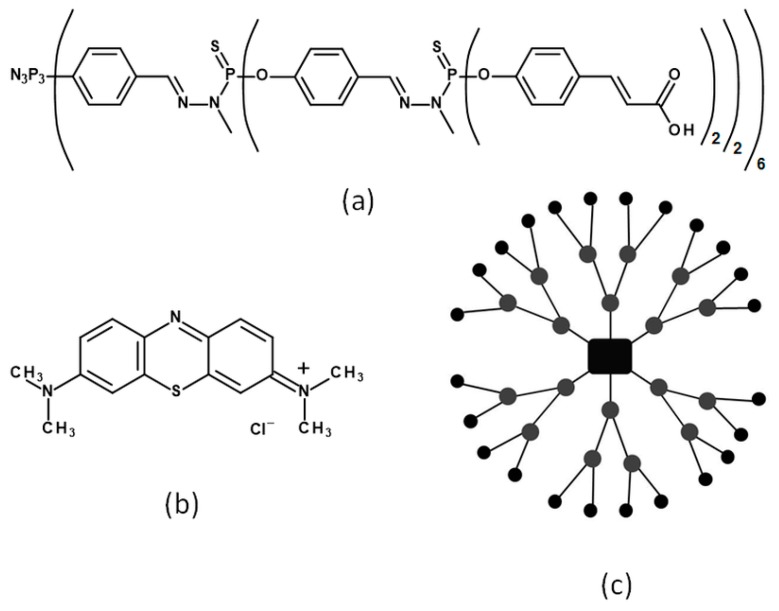
The structures of the compounds used in this study—(**a**) linear structure of anionic phosphorus dendrimer 1an; (**b**) methylene blue; (**c**) schematic representation of the second generation anionic dendrimer structure.

Phosphorus dendrimer of the second generation with 24 carboxyl terminal groups (1an) was synthesized by the Laboratoire de Chimie de Coordination du CNRS [31,32]. Methylene blue (MB), MTT (3-(4,5-dimethyl-2-thiazolyl)-2,5-diphenyl-2*H*-tetrazolium bromide), propidium iodide, fetal bovine serum, trypsin-EDTA solution, and penicillin-streptomycin solution were obtained from Sigma-Aldrich (St. Louis, MO, USA). Dulbecco’s phosphate-buffered saline with no calcium and no magnesium (DPBS) was purchased from Biowest (Riverside, MO, USA). Culture medium 154 CF was purchased from Gibco^®^ (ThermoFisher Scientific, Waltham, MA, USA). Singlet oxygen sensor green (SOSG) and 2′,7′-dichlorodihydrofluorescein diacetate (H2DCFDA) was purchased from Molecular Probes™ (ThermoFisher Scientific, Waltham, MA, USA). Trypan blue stain was obtained from Invitrogen™ (ThermoFisher Scientific, Waltham, MA, USA). Chelex^®^ 100 resin was obtained from Bio-Rad (Hercules, CA, USA). Annexin V-fluorescein isothiocyanate (FITC) and binding buffer was obtained from BD Biosciences (San Jose, CA, USA). Dimethyl sulphoxide (DMSO) was purchased from POCH (Gliwice, Poland). The structures of 1an and MB are presented in Scheme 1.

### 4.2. Cell Culture

As an in vitro model of basal cell carcinoma we used three BCC lines (ASZ, BSZ, and CSZ) [24] that were kindly gifted by Dr. Ervin Epstein (Children’s Oakland Research Institute, Oakland, CA, USA). Cells were grown in 154-CF medium (Gibco, ThermoFisher Scientific, Waltham, MA, USA) supplemented with 2% chelexed, heat-inactivated FBS, antibiotics, and 0.05 mM calcium. Cells were incubated at 37 °C in a humidified incubator under a 5% CO_2_ atmosphere and cultured in T75 culture flasks. Cells were subcultured every two or three days. For all experiments cells were harvested using 0.25% (*w*/*v*) trypsin–0.03% (*w*/*v*) EDTA solution and counted using a trypan blue exclusion assay. We used a Countess Automated Cell Counter (Invitrogen, ThermoFisher Scientific, Waltham, MA, USA) for counting the cells.

### 4.3. Singlet Oxygen Production Assay

In order to determinate the singlet oxygen formation by MB and MB-1an complex, we used singlet oxygen sensor green (SOSG) which is a comercially available probe and is highly selective for ^1^O_2_. We used SOSG in 1 µM concentration. The solutions of MB and MB-1an were prepared in 10 mM DPBS buffer. We used three different concentrations of MB: 1.0, 2.5, and 5.0 µM. A molar ratio of the complex was 5:1 (MB:1an). Respectively, the 1an dendrimer concentrations in a complex were 0.2, 0.5, and 1.0 µM. Following the sample preparation, a 100 µL of solution was transferred to a 96-well black plate. All measurements were recorded using fluorescence microplate reader (Fluoroskan Ascent FL, ThermoFisher Scientific, Waltham, MA, USA). The excitation and emission filters were 500 nm and 538 nm, respectively. The plates were shaken prior to every measurment. The first measurement was recorded without SOSG in order to determine whether MB or MB-1an exhibit emission in this region. The compounds did not show any emission in this region. After the first measurement, SOSG was added to each well and the fluorescence of SOSG without irradiation was checked. Next, the plate was immediately placed under the Q.Light Pro Unit lamp equipped with a filter emitting red light from 620 to 780 nm. The distance from the light source was 20 cm. The power density of the light source was 40 mW/cm^2^ and the light dose of 2.4 J/cm^2^ per minute. The samples were irradiated and the fluorescence was measured in the course of the irradiation time, from 1 to 60 min.

### 4.4. Cellular Uptake

For the determination of cellular uptake, ASZ, BSZ, and CSZ lines were seeded in 96-well plates at density 3 × 10^4^ cells per well. To allow cells to attach, the plates were incubated for 24 h in a humified incubator (New Brunswick, Eppendorf, Hamburg, Germany) under 5% CO_2_ atmosphere. Next, the culture medium was removed and MB and MB-1an complex were added to wells in DPBS. The concentration of MB was 5 µM, and due to the ratio of the complex being 5:1 (MB:1an), the concentration of the dendrimer in the complex was 1 µM. The cells were incubated with compounds for up to 7 h. Next, the solutions of the compounds were removed, cells were washed with DPBS and lysed using 30 µL of 1% Triton X-100 in 4 °C. Then, the absorbance at 664 nm was measured using a microplate reader (PowerWave HT Microplate Spectrophotometer, Biotek, Winooski, VT, USA). The plates were gently shaken prior to measurements. The cellular uptake was determined as the concentration of MB basing on the standard curve for MB. As the controls, the absorbance of pure solutions of MB and MB-1an were used.

### 4.5. Cytotoxicity Assay and the Irradiation Protocol

Cells were seeded in 96-well plates. The density of the cells was equal to 3 × 10^4^ cells per well. Next, the cells were incubated for 24 h in 37 °C and 5% CO_2_. Following the incubation, the culture medium was replaced with solutions of free photosensitizer methylene blue (MB) and complex (MB-1an) in DPBS. A molar ratio of the complex was 5:1 (MB:1an). For the untreated control group, DPBS was added to the wells. Methylene blue was used at concentrations 1, 2.5, 5, and 10 μM. Cells were incubated with free photosensitizer and complex for 5 h. Then, cells were exposed to visible light irradiation using a broad-spectrum light source (Q.Light Pro Unit, Q.Products AG, Rorschach, Switzerland). For photosensitization of MB molecules we used a filter emitting red light from 620 nm to 780 nm. The distance from the light source was 20 cm. The power density of the light source was 40 mW/cm^2^ and the total light dose was 72 J/cm^2^. After 30 min of irradiation, cells were incubated for 1 h and DPBS solutions were replaced with fresh culture medium. Then cells were incubated for another 24 h and cell viability was measured using MTT assay. The culture medium was discarded from the wells and the cells were rinsed with DPBS buffer. Then, 50 µL of MTT (0.5 mg/mL) was added to each well. After 3 h of incubation, the MTT was discarded and formazan crystals were dissolved using 100 µL of DMSO. The absorbance was read using PowerWave HT Microplate Spectrophotometer (Biotek, Winooski, VT, USA) at 570 nm. The dark toxicity of dendrimers, pure MB and complexes was evaluated after incubation without irradiation.

### 4.6. Detection of Apoptotic and Necrotic Cells after the Irradiation

In order to detect apoptotic and necrotic cells we used Annexin V-fluorescein isothiocyanate (FITC)/propidium iodide (PI) staining. The double staining allows the detection of apoptotic cells upon translocation of phosphatydylserine to the outer layer of the plasma membrane because of the high affinity of Annexin V to this phospholipid. Apoptotic cells stained with Annexin V-FITC exclude PI, while necrotic cells are permeable to PI. This staining allows the differentiation between viable, apoptotic, and necrotic cell populations. Cells were seeded in 12-well plates at the density of 2.5 × 10^5^ cells per well and incubated for 24 h. Then, MB and MB-1an were added where the concentration of MB and 1an was 2.5 µM and 0.5 µM, respectively. After 5 h incubation, cells were irradiated as in the previous experiments. Next, cells were washed with DPBS and detached. After centrifugation at 2500 rpm, cells were suspended in 200 µL of binding buffer (delivered by the producer) containing 4 µL of Annexin V-FITC and 0.2 µL of PI. Samples were incubated for 15 min at 4 °C in the dark. Fluorescence intensity was measured with a Becton Dickinson LSR II flow cytometer (BD Biosciences, San Jose, CA, USA). As positive controls, apoptotic and necrotic cells were used. Apoptosis was induced using 80 µM camptothecin and necrosis was used using frozen ethanol (data not shown).

### 4.7. Reactive Oxygen Species Production

The intracellular production of ROS was investigated by the H2DCFDA molecular probe (2′,7′-dichlorodihydrofluorescein diacetate, Molecular Probes™, ThermoFisher Scientific, Waltham, MA, USA). The detection method is based on the two reactions of the H2DCFDA. First, the acetate groups of H2DCFDA are cleaved by intracellular esterases. Next, upon oxidation, non-fluorescent H2DCF is converted to a highly fluorescent form—dichlorofluorescein (DCF). The intensity of DCF fluorescence is proportional to the amount of intracellular reactive oxygen species. Briefly, to the 96 well black plates containing 3 × 10^4^ cells per well, solutions of MB, MB-1an and 1an were added. The concentration of free and complexed MB was 5 µM and the 1an concentration was 1 µM. DPBS was used as a control sample. Cells were incubated with compounds for 5 h at 37 °C in a humified atmosphere containing 5% CO_2_. Next, the cells were washed with DPBS and a solution of 2 µM H2DCFDA in DPBS was added to each well. Cells were incubated for 15 min in dark. After the probe solution was discarded, the cells were washed with DPBS and, next, 100 µL of DPBS was added. Next, a 485/530 nm fluorescence of DCF was measured as a background using a BIOTEK PowerWave HT Microplate reader (Biotek). Then cells were irradiated for 30 min, and after that the fluorescence was measured. Every measurement was corrected by subtraction of the background fluorescence intensity.

### 4.8. Statistical Analysis

All data are presented as a mean ± standard deviation. One-way analysis of variance (ANOVA) was used for multiple comparisons and followed by post-hoc Tukey test. A *p*-value of < 0.05 was considered as statistically significant. The statistical data was analyzed using Sigma Plot version 12.5 (Systat Software, San Jose, CA, USA).

## Supplementary Materials

Supplementary materials are available online.
