# Supplementary material for: Complexing Methylene Blue with Phosphorus Dendrimers to Increase Photodynamic Activity
Source: Molecules. 2017 Feb 23;22(3):345. doi: 10.3390/molecules22030345 (PMC6155338; doi:10.3390/molecules22030345)
Supplement: Supplementary file 1 [file molecules-22-00345-s001.pdf]

Complexing Methylene Blue with Phosphorus Dendrimers to Increase Photodynamic Activity

Monika Dabrzalska, Anna Janaszewska, Maria Zablocka, Serge Mignani, Jean Pierre Majoral and Barbara Klajnert-Maculewicz

Table S1. Viability (% of control) of ASZ cell line after the treatment with MB and MB-1an without irradiation (dark toxicity).

MB [μM]	MB	MB-1an
1.0	87.62 ± 5.30	79.33 ± 14.14
2.5	82.88 ± 6.50	79.34 ± 8.51
5.0	82.39 ± 3.21	78.01 ± 6.13
10.0	68.14 ± 8.56	68.31 ± 13.07

Table S2. Viability (% of control) of BSZ cell line after the treatment with MB and MB-1an without irradiation (dark toxicity).

MB [μM]	MB	MB-1an
1.0	86.85 ± 0.29	92.36 ± 10.24
2.5	82.83 ± 0.85	94.55 ± 8.56
5.0	74.49 ± 1.57	87.27 ± 4.74
10.0	54.87 ± 5.19	62.31 ± 11.62

Table S3. Viability (% of control) of CSZ cell line after the treatment with MB and MB-1an without irradiation (dark toxicity).

MB [μM]	MB	MB-1an
1.0	93.03 ± 3.83	92.82 ± 7.78
2.5	84.47 ± 7.28	87.61 ± 8.39
5.0	81.79 ± 10.30	74.17 ± 11.05
10.0	74.19 ± 10.98	61.74 ± 4.36

Table S4. Viability (% of control) of ASZ, BSZ and CSZ cell lines after the treatment with 1an without irradiation.

1an [μM]	ASZ	BSZ	CSZ
0.2	88.84 ± 5.40	88.88 ± 12.41	90.58 ± 3.47
0.5	88.36 ± 5.85	96.04 ± 11.33	84.74 ± 2.45
1.0	90.85 ± 8.92	93.00 ± 2.65	80.90 ± 2.21
2.0	78.58 ± 3.66	73.77 ± 14.22	65.55 ± 4.66

Table S5. Viability (% of control) of ASZ cell line after the treatment with different concentrations of MB and MB-1an after the 30 min irradiation.

MB [μM]	MB	MB-1an
1.0	83.4 ± 2.04	79.22 ± 3.21
2.5	83.71 ± 1.13	70.91 ± 4.49
5.0	80.55 ± 6.91	20.21 ± 5.07
10.0	59.55 ± 1.57	11.49 ± 5.50

Table S6. Viability (% of control) of BSZ cell line after the treatment with different concentrations of MB and MB-1an after the 30 min irradiation.

MB [μM]	MB	MB-1an
1.0	90.06 ± 4.57	79.29 ± 6.06
2.5	76.17 ± 2.24	51.74 ± 5.33
5.0	51,35 ± 4.13	17.41 ± 3.71
10.0	29.37 ± 2.31	8.68 ± 3.35

Table S7. Viability (% of control) of CSZ cell line after the treatment with different concentrations of MB and MB-1an after the 30 min irradiation.

MB [μM]	MB	MB-1an
1.0	86.84 ± 11.15	80.65 ± 6.17
2.5	83.48 ± 8.86	69.55 ± 6.79
5.0	65.68 ± 4.25	24.03 ± 12.83
10.0	54.72 ± 5.64	9.95 ± 5.57
